# Glucagon-Like Peptide-1 Is Associated With Systemic Inflammation in Pediatric Patients Treated With Hematopoietic Stem Cell Transplantation

**DOI:** 10.3389/fimmu.2021.793588

**Published:** 2021-12-08

**Authors:** Maria Ebbesen, Hannelouise Kissow, Bolette Hartmann, Katrine Kielsen, Kaspar Sørensen, Sara Elizabeth Stinson, Christine Frithioff-Bøjsøe, Cilius Esmann Fonvig, Jens-Christian Holm, Torben Hansen, Jens Juul Holst, Klaus Gottlob Müller

**Affiliations:** ^1^ Department of Pediatrics and Adolescent Medicine, University Hospital Rigshospitalet, Copenhagen, Denmark; ^2^ Novo Nordisk Foundation Center for Basic Metabolic Research, Faculty of Health and Medical Sciences, University of Copenhagen, Copenhagen, Denmark; ^3^ Department of Biomedical Sciences, Faculty of Health and Medical Sciences, University of Copenhagen, Copenhagen, Denmark; ^4^ Institute for Inflammation Research, Center for Rheumatology and Spine Diseases, University Hospital Rigshospitalet, Copenhagen, Denmark; ^5^ The Children’s Obesity Clinic, Accredited European Centre for Obesity Management, Department of Pediatrics, Copenhagen University Hospital Holbæk, Holbæk, Denmark; ^6^ Department of Pediatrics, Kolding Hospital a Part of Lillebælt Hospital, Kolding, Denmark

**Keywords:** hematopoietic stem cell transplantation, high-dose chemotherapy, glucagon-like peptide-1, toxicity, systemic inflammation, pediatrics, growth factors

## Abstract

Patients undergoing allogeneic hematopoietic stem cell transplantation (HSCT) are challenged with severe side effects, which are propagated by mucosal barrier disruption, and the related microbial translocation and systemic inflammation. Glucagon-like peptide-1 (GLP-1), a well-known incretin hormone, possesses anti-inflammatory properties and promotes regeneration of damaged intestinal epithelium in animal studies. We hypothesized that the immense inter-individual variation in the degree of mucosal damage and systemic inflammation, seen after HSCT is influenced by endogenous GLP-1 and could be related to acute post-transplant complications. In this prospective study we measured serial weekly fasting plasma GLP-1, along with C-reactive protein (CRP), and citrulline in 82 pediatric patients during allogeneic HSCT together with a fasting plasma GLP-1 in sex- and age-matched healthy controls. Overall, GLP-1 levels were increased in the patients during the course of HSCT compared with the controls, but tended to decrease post-transplant, most pronounced in patients receiving high-intensity conditioning regimen. The increase in CRP seen in the early post-transplant phase was significantly lower from day +8 to +13 in patients with GLP-1 above the upper quartile (>10 pmol/L) at day 0 (all *P* ≤ 0.03). Similar findings were seen for peak CRP levels after adjusting for type of conditioning (-47.0%; 95% CI, -8.1 – -69.4%, *P* = 0.02). Citrulline declined significantly following the transplantation illustrating a decrease in viable enterocytes, most evident in patients receiving high-intensity conditioning regimen. GLP-1 levels at day 0 associated with the recovery rate of citrulline from day 0 to +21 (34 percentage points (pp)/GLP-1 doubling; 95% CI, 10 – 58pp; *P* = 0. 008) and day 0 to day +90 (48 pp/GLP-1 doubling; 95% CI, 17 – 79pp; *P* = 0. 004), also after adjustment for type of conditioning. This translated into a reduced risk of acute graft-versus-host disease (aGvHD) in patients with highest day 0 GLP-1 levels (>10 pmol/L) (cause-specific HR: 0.3; 95% CI, 0.2 – 0.9, *P* = 0.02). In conclusion, this study strongly suggests that GLP-1 influences regeneration of injured epithelial barriers and ameliorates inflammatory responses in the early post-transplant phase.

## Introduction

Allogeneic hematopoietic stem cell transplantation (HSCT) in children and adolescents is challenged by adverse events, which to a large extent are related to toxic reactions in the gastrointestinal tract (1). Oral and gastrointestinal mucositis are reported in up to 90 – 100% of patients during HSCT with myeloablative conditioning (2–4), and substantial evidence indicates that intestinal toxicity induces severe systemic inflammation and translocation of bacterial products, leading to increased risk of acute graft-versus-host-disease (aGvHD), invasive infections, multi-organ failure and treatment-related mortality (5–8).

The susceptibility of the patients to develop these complications is highly variable, which opens a window for personalized treatment, but further progress is hampered by the absence of predictive biomarkers. Moreover, there is no effective treatment preventing severe mucositis and the current treatment is symptomatic, based on parenteral hydration and nutrition, pain relief by morphine and use of broad-spectrum antibiotics.

However, sustained proliferation of intestinal epithelium is known to be elemental in healing of mucositis and maintenance of the intestinal barrier and involves growth factors produced in the gut (9, 10). Glucagon-like peptide-1 (GLP-1) is a peptide hormone secreted from enteroendocrine L-cells following enteral food intake, and is well-known for being essential in regulating blood glucose by the stimulation of insulin secretion (11, 12). Additionally, increased secretion of GLP-1 is seen after chemotherapy-induced intestinal injury in rodents (13, 14), and elevated plasma levels are observed in humans after chemotherapy (15) and during gut ischemia (16). Indeed, GLP-1 has intestinotrophic effects sustaining the integrity of intestinal mucosal barrier in animal studies (17, 18). Administration of GLP-1 analogs can ameliorate chemotherapy-induced intestinal injury (13), while ablation of L-cells in mice has led to severe mucositis as well as insufficient intestinal healing after chemotherapy (14, 19). In addition, GLP-1 acts as a direct anti-inflammatory mediator locally through GLP-1-receptors expressed on intestinal intraepithelial lymphocytes (20, 21).

In the present study, we measured fasting GLP-1 plasma levels in pediatric patients undergoing HSCT from before the start of conditioning and during the early post-transplant period to determine possible associations between GLP-1 and intestinal damage, measured by citrulline levels, systemic inflammation and post-HSCT complications.

## Materials and Methods

### Study Population

In this prospective population-based study, 82 children and adolescents (1–18 years of age) undergoing their first allogeneic HSCT were consecutively recruited at University Hospital Rigshospitalet, Copenhagen, Denmark, from March 2015 to November 2019. This patient cohort has previously been described in a different context (22).

Conditioning groups were defined as 1) high-intensity myeloablative conditioning (total body irradiation (TBI) 12Gy + etoposide, busulfan + cyclophosphamide, or busulfan + thiotepa + fludarabine) and 2) low-intensity myeloablative conditioning (other fludarabine-based regimens or cyclophosphamide + TBI 2Gy) (23) (**Table 1**).

**Table 1:** Diagnoses and transplantation modalities, n = 82.

Pre-transplant diagnoses	
**Disease at transplantation, no. of patients (%)**	
Acute myeoloid leukemia	13 (16)
Acute lymphoblastic leukemia	21 (26)
Juvenile myelo-monocytic chronic leukemia	2 (2)
Myelodysplastic syndrome	10 (12)
Other malignancies	2 (2)
Severe aplastic anemia	6 (7)
Immunodeficiency	13 (16)
Other non-malignant diseases	15 (18)
**Transplantation data**	
**Donor type, no. of patients (%)**	
HLA-identical siblings	27 (33)
HLA-matched unrelated donors (10/10 match)	44 (54)
HLA-mismatched unrelated donors (9/10 or 8/10 match)	11 (13)
**Stem cell source, no. of patients (%)**	
Bone marrow stem cells	73 (89)
Peripheral blood stem cells, G-CSF mobilized	4 (5)
Umbilical cord blood	5 (6)
**Conditioning regimen, no. of patients (%)**	
(1)High-intensity myeloablative conditioning:	
TBI 12 Gy + etoposide	14 (17)
BU + CY	21 (26)
BU + thiotepa + FLU	7 (9)
(2)Low-intensity myeloablative conditioning:	
FLU + treosulfan +/- thiotepa	23 (28)
FLU + CY	6 (7)
FLU + BU	8 (10)
TBI 2 Gy + CY	3 (4)
**Sex mismatch (female donor to male recipient), no. (%)**	17 (21)

BU, busulfan; CY, cyclophosphamide; FLU, fludarabine; G-CSF, granulocyte colony-stimulating factor; HLA, human leucocyte antigen; TBI, total body irradiation.

### Control Cohort

A control cohort matched by sex and nearest age option with a patient/control frequency ratio of 1:5 (N = 410 controls, **Table 2**) was included from a population-based cohort of Danish/North-European children and adolescents without obesity and diabetes, 6-18 years of age (N = 2,266), enrolled in The Danish Childhood Obesity Data- and Biobank from 2009 - 2019 and previously described (24, 25).

**Table 2:** Age and sex in the HSCT-patients and the sex- and age-matched control group, frequency ratio 1:5.

	HSCT-patients	Control cohort	*P*-value
**No. of individuals**	82	410	
**Age (median [IQR])**	8.84 [5.67, 13.28]	8.82 [6.98, 13.06]	0.2
**Sex, M (%)**	43 (52.4)	236 (57.6)	0.5

### Blood Samples for Laboratory Analyses

Blood samples were collected at 6 AM at the following time points: before start of the conditioning regimen, at the day of transplantation (day 0), and at days +7, +14 and +21 post-transplantation. The control cohort had a single venous blood sample collected between 7 and 9 AM, following an overnight fast (26). EDTA anticoagulated blood was centrifuged up to two hours after collection and plasma was isolated and stored at -80°C.

### Quantification of GLP-1

The plasma concentration of total GLP-1 in both patients and controls was measured in duplicates using a GLP-1 ELISA kit (Mercodia, Uppsala, Sweden) according to the manufacturer’s instructions (27). Both active GLP-1 (7-36) amide and the degraded isoform GLP-1 (9-36) amide were measured and reflects the secretion of GLP-1 because amidated isoforms of GLP-1 are highly predominant in humans (28). Measurement range was 0.9 to 940 pmol/L.

### Citrulline

As a marker of enterocyte damage, plasma citrulline was measured at the same time points as GLP-1 and at follow-up day +90 post-HSCT. A Waters Acquity™ Ultra-Performance Liquid Chromatography system with a Tandem Quadrupole detector was used for the analysis (29).

### CRP

All patients had CRP monitored daily during the first three weeks after transplantation. When more than one measurement per day was available, the mean was calculated to represent the CRP level of that day. CRP_max_ was defined as maximum CRP value from day +1 to day +21. CRP was analyzed using Modular P Module (Roche, Basel, Switzerland) (upper normal limit, 10 mg/L) at the Department of Clinical Biochemistry, University Hospital Rigshospitalet. Blood cultures were routinely collected on all patients with fever and results were registered from day -14 to day +30.

### Statistical Analyses

A mixed model with a compound symmetry covariance matrix was used to investigate associations of GLP-1 and citrulline over time with patient-specific characteristics and the association between CRP over time and dichotomized GLP-1 level at day 0. GLP-1 and CRP_max_ were log-transformed due to their skewness.

Correlation analyses were performed using Spearman’s rank order correlation analysis. The Mann-Whitney U-test or the Kruskal–Wallis univariate test were used for comparisons between groups. Simple and multiple linear regression models were used to determine the association between GLP-1 and CRP_max_. All potential risk factors listed in **Tables 1**, **2** were tested in univariate analyses and included in the multivariate model if they showed statistically significant associations with outcome variables and/or GLP-1 as indicated under results. Interaction between GLP-1 and conditioning group stratification on CRP_max_ was included as a covariate in the initial multivariate model and tested with ANOVA. Logistic simple and multiple regressions were used to determine variables associated with occurrence of aGvHD and sinusoidal obstruction syndrome (SOS). Cumulative incidences, cause-specific Cox regression models and Gray’s test (30) were used to estimate the risk of aGvHD.

Statistical significance was defined as a two-sided P < 0.05. All statistical analyses were performed using R statistical software version 3.6.1 [R Foundation for Statistical Computing, Vienna, Austria (31)] and RStudio version 1.2.1335 (RStudio, Boston, MA).

### Ethics Statement

The study was approved by Capital Region of Denmark’s Ethical Committee (H-7-2014-016) and conducted in accordance with the Declaration of Helsinki. Written informed consent was obtained from all included patients and/or their legal guardians.

## Results

Clinical characteristics of patients and controls are presented in **Tables 1**, **2**. Before start of conditioning, 44 out of the 82 included patients had a fasting sample available for GLP-1 measurement. These patients did not differ from the rest of the cohort regarding sex, age, or diagnosis. At the remaining time points all patients had fasting GLP-1 measurements available.

### GLP-1 in Patients and Healthy Controls

**Figure 1:**
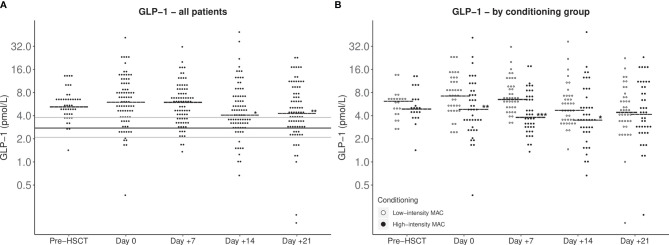
GLP-1 fasting plasma levels during pediatric HSCT from before conditioning until day +21 post-HSCT. Horizontal short lines: Median GLP-1. **(A)** All included patients. Consistent lines: Median (black), lower and upper quartile (grey) for fasting GLP-1 for healthy control cohort. Statistical evaluation indicates increased GLP-1 levels of HSCT pediatric patients compared with the control cohort at all time points (*P* < 0.001). A mixed model analysis showed GLP-1 levels at day +14 and day +21 to be lower than day 0 GLP-1 levels (*P < 0.05; **P < 0.005). **(B)** Patients stratified by conditioning group. Patients treated with high-intensity conditioning regimens had generally lower post-HSCT GLP-1 levels than patients treated with low-intensity conditioning evaluated with a mixed model analysis (*P < 0.05; **P < 0.005; ***P < 0.001).

Median fasting GLP-1 levels in the patients were higher than in the controls at all time points but decreased during the first weeks of the transplantation, most pronounced in patients receiving high-intensity myeloablative conditioning (**Figure 1**).

### Inflammation and GLP-1

**Figure 2:**
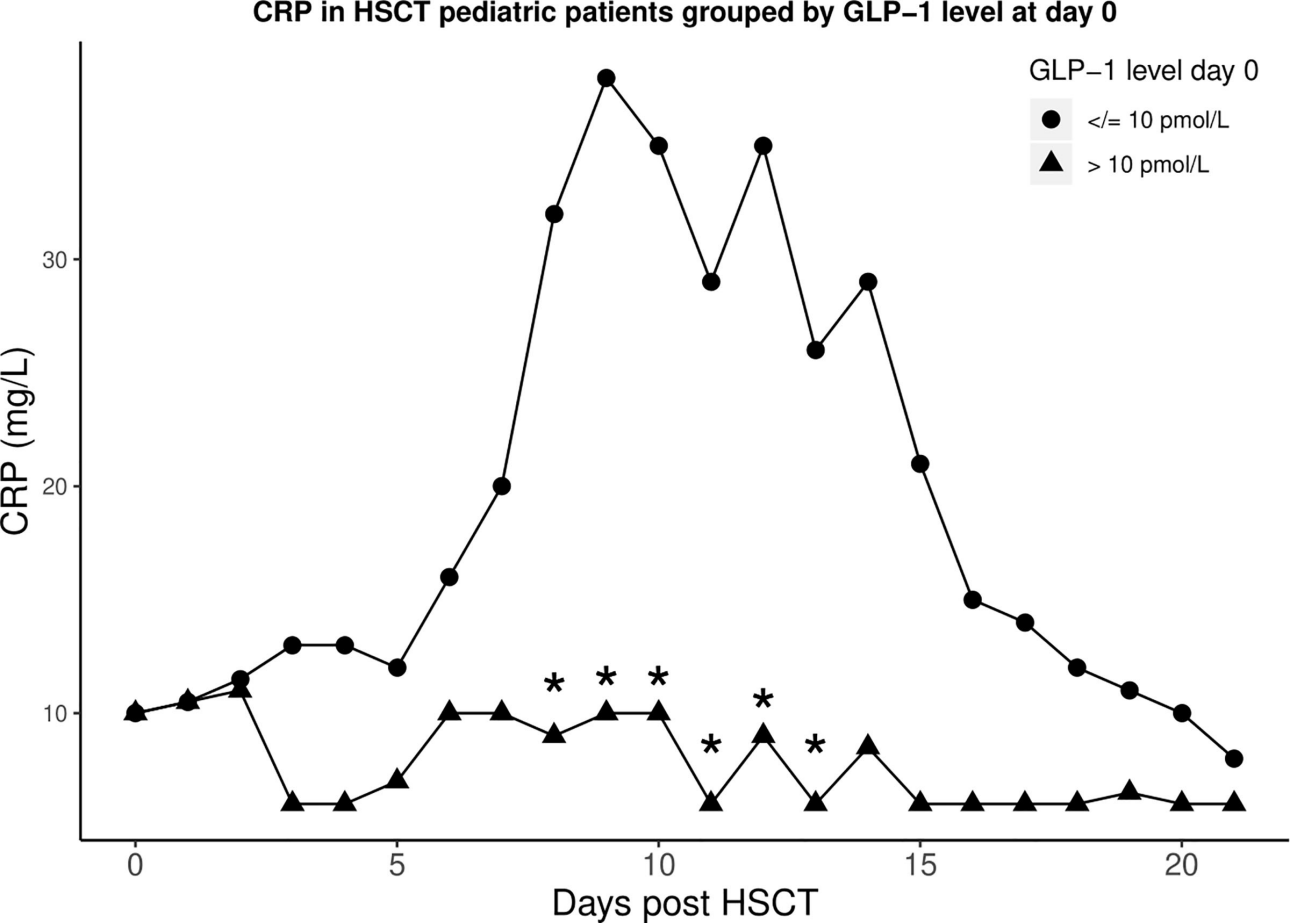
Median CRP levels after HSCT in two groups stratified by GLP-1 level at day 0 [≤10 pmol/L (circles) and >10 pmol/L (triangles)]. *CRP levels significantly differ between the two groups (all *P* ≤ 0.03).

To explore potential anti-inflammatory protective effects of GLP-1, we investigated associations between GLP-1 and CRP. CRP increased significantly during the course of transplantation reaching a maximum at day +9. Peak CRP levels were significantly higher in patients receiving high-intensity vs low-intensity conditioning (median CRP_max_ 35 vs. 89 mg/L (*P* < 0.001) but were not associated with other patient- and transplant-related characteristics. Stratifying patients in two groups according to GLP-1 levels at day 0, patients with GLP-1 levels in the upper quartile (>10 pmol/L) had significantly lower CRP_max_ levels (-56%; 95% CI, -20– -76%, *P* = 0.007). This association remained significant after adjusting for type of conditioning (-47.0%; 95% CI, -8.1 – -69.4%, *P* = 0.02). There was no interaction between GLP-1 levels and conditioning group on CRP_max_. Likewise, when looking at daily CRP measurements post-HSCT, GLP-1 levels above the upper quartile at day 0 were associated with lower levels of CRP from day +8 to day +13 (all *P* ≤ 0.03) (**Figure 2**).

### Citrulline and GLP-1

**Figure 3:**
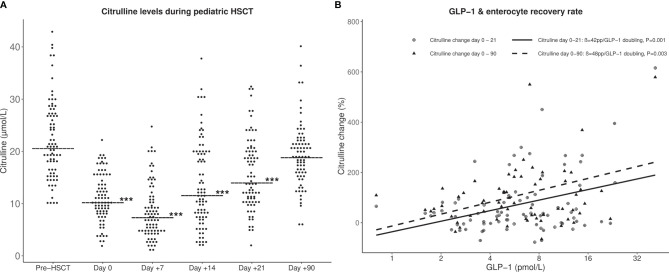
**(A)** Citrulline levels during HSCT from before conditioning until 90 days post-HSCT. Horizontal line: median citrulline. ***Citrulline levels significantly lower than pre-HSCT levels (*P* < 0.001). **(B)** Associations between GLP-1 levels at day of transplant (day 0) and relative citrulline increase from day 0 to day +21 (solid line) and from day 0 to day +90 (dashed line). P-values by simple linear regression models. pp, percentage point increase in citrulline.

The systemic inflammatory response during the aplastic phase of HSCT has to a large extent been related to disintegration of the intestinal mucosal barrier, which can be effectively monitored by plasma citrulline, being a marker of the total population of viable enterocytes (32). Citrulline levels decreased significantly after conditioning therapy reaching nadir at day +7, coinciding with the timepoint of maximum CRP levels. At day +90, citrulline had again risen close to pre-HSCT levels [median (IQR): 19.0 (16.0 - 22.0) vs 20.7 (16.5 - 27.2) μmol/L] (**Figure 3A**). The citrulline decrease was most pronounced in patients receiving high-intensity myeloablative conditioning and with a delayed recovery compared with those undergoing low-intensity conditioning (significantly lower levels at day +14 and +21, but comparable levels at day +90).

To further investigate an effect of GLP-1 on epithelial repair, associations between GLP-1 levels and the rate of citrulline recovery were explored. GLP-1 levels at day 0 were significantly associated with the relative increase in citrulline levels from day 0 to +21 (42 percentage point (pp)/GLP-1 doubling; 95% CI, 18 – 66pp; *P* = 0. 001) and from day 0 to +90 (48 pp/GLP-1 doubling; 95% CI, 17 – 77pp, *P* = 0. 003) (**Figure 3B**). This remained significant in multivariate analyses adjusting for conditioning regimen (34; 95% CI, 10 – 58; *P* = 0. 008, and 48 pp/GLP-1 doubling; 95% CI, 17 – 79pp; *P* = 0. 004, respectively).

### Clinical Outcomes

**Figure 4:**
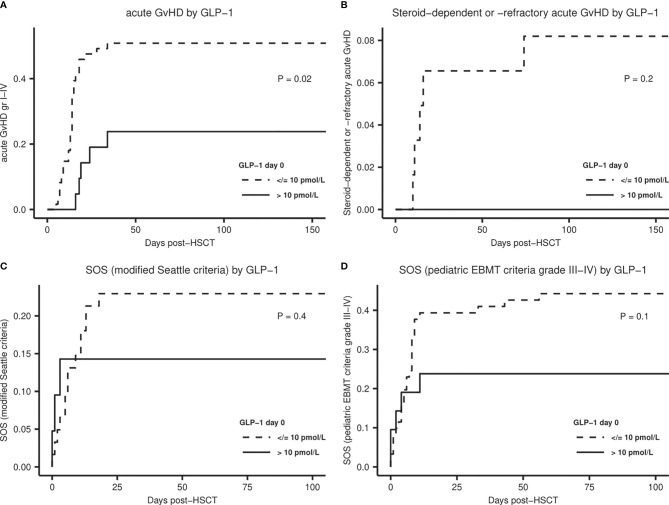
Cumulative incidence plots for acute treatment-related complications stratified by GLP-1 level at day 0 (≤10 pmol/L). **(A)** Acute GvHD, **(B)** Steroid-dependent or steroid-refractory acute GvHD, **(C)** Sinusoidal obstruction syndrome (SOS) diagnosed according to the modified Seattle criteria. **(D)** SOS diagnosed according to the pediatric EBMT criteria, severity grade III-IV. P-values by Gray’s test.

A total of 35 patients (43%) developed acute graft-versus-host disease (aGvHD) with onset at median day +14 (range: +5 to +34); grades III–IV aGvHD were seen in nine patients (11%). Patients with day 0 GLP-1 levels above the upper quartile showed a reduced risk of developing aGvHD (cause-specific HR: 0.3; 95% CI, 0.2-0.9, *P* = 0.02) (**Figure 4A**). In a multivariate analysis adjusting for the conditioning regimen this remained significant (HR: 0.4; 95% CI, 0.2 – 1.0, *P* = 0.04). Three and two patients developed steroid-dependent and steroid-refractory aGvHD, respectively. Although this limited number did not allow for conclusions regarding association with GLP-1 levels we noticed that none of these five patients had GLP-1 levels in the upper quartile (**Figure 4B**).

SOS was diagnosed in 17 (21%) of the patients according to the modified Seattle criteria (33) and in 41 (50%) according to the pediatric EBMT criteria (34), with severity grades III–IV in 32 (39%) of the patients. The frequency of SOS was not related to pre-transplant diagnosis. CRP_max_ was significantly associated with risk of SOS, also in multivariate analyses, both according to the modified Seattle criteria (OR = 2.1 per CRP_max_ doubling; 95% CI, 1.3 – 3.9; *P* = 0. 009) and to the pediatric EBMT grade III-IV (OR = 1.7 per CRP_max_ doubling; 95% CI, 1.1 – 2.7; *P* = 0.02). Cumulative incidence plots showed a tendency to lower incidences of SOS over time in patients with GLP-1 levels above the upper quartile (**Figures 4C, D**), even though not reaching statistical significance.

Ten patients (12%) developed bacteremia at median day +22 (0 to +84) post-HSCT. We were not able to make any conclusions regarding associations between bacteremia and GLP-1 or citrulline levels, most likely due to the limited incidence.

Median follow-up time was 2.0 years (1.0 – 4.7) from transplantation. Three patients died after HSCT, two of these due to treatment-related complications and one patient did not engraft. Of the 48 patients transplanted for a malignant disease, two patients relapsed. These limited numbers did not allow conclusions regarding prediction of survival by GLP-1 levels.

## Discussion

In the present study, we investigated fasting levels of GLP-1 during the course of allogeneic HSCT to achieve new insights into the potentially protective effects of GLP-1 on treatment-related toxicity.

Although increased before start of conditioning in comparison with sex- and age-matched healthy children and adolescents, GLP-1 levels tended to decline during the course of transplant, most pronounced in patients receiving high-intensity conditioning therapy. A high GLP-1 level at day 0, the start of the toxic-aplastic phase, was associated with less systemic inflammation and with a faster recovery of enterocytes in terms of citrulline increase, even within the same conditioning group, indicating a protective and restorative effect of GLP-1. These findings translated into a reduced risk of aGvHD in patients with high GLP-1 levels.

Various animal studies have substantiated the intestinotrophic effects of GLP-1, which have been found comparable in size with the well-known effects of the closely related and co-secreted GLP-2 (13, 17, 19). Endogenous GLP-1 has been shown to be important for intestinal mucosal recovery in mice treated with chemotherapy (13, 14). The underlying mechanism of the intestinotrophic effects of both GLP-1 and GLP-2 are poorly understood. A signal interaction where GLP-1 potentiates the trophic effect of GLP-2 has been suggested (35), and co-treatment with the two peptides showed superior effects in recovery of chemotherapy-induced mucositis in mice compared with GLP-1 or GLP-2 monotherapy (14). Additional protective effects of GLP-1 have been suggested by studies demonstrating GLP-1-induced secretion of antimicrobial peptides from Paneth cells (36) and activated production of the barrier-protective mucus layer by Brunner’s glands (37). Furthermore, GLP-1 seems capable of directly modulating local inflammation in the gut by its interaction with intestinal epithelial lymphocytes leading to reduced pro-inflammatory cytokine secretion (20). A mucosal restorative effect of GLP-1 was observed in the present study, as GLP-1 levels at day of HSCT positively associated with post-HSCT citrulline increase, reflecting accelerated recovery of functioning enterocytes in patients with higher GLP-1. Together, the pleiotropic effects of GLP-1 could potentially contribute to the protection against a harmful systemic inflammatory response following the toxic impact on the intestinal epithelium and its down-stream effects in terms of non-infectious organ toxicities. Our data indicates that the ability to maintain GLP-1 levels after chemotherapy is important for protecting the gut against the damaging effects of the cytotoxic treatment and the resulting severe systemic inflammatory response. In other words, we suggest that GLP-1 may be defined as a factor enhancing “tissue tolerance”, which has appeared to be critical for disease severity in general (38). The demonstrated association between GLP-1 levels and aGvHD, even within the same group of conditioning regimen, in the present study, lend support to a protective role of GLP-1 against treatment-related complications and in line with our findings, a recent study by Norona et al. indicated L cells being a target of aGvHD and showed that lower numbers of L-cells were associated with increased mortality risk in adult patients with aGvHD (39). Despite a significant association between peak CRP levels and SOS, we were unable to demonstrate any significant association between GLP-1 and SOS, most likely due to lack of power in combination with the complex mechanisms behind SOS involving several other predisposing factors.

The factors that determine the rate of GLP-1 secretion and the resulting circulating GLP-1 levels during intestinal traumas are not fully understood. Indeed, a number of studies including both animal and clinical studies have suggested that the gut may respond to cytotoxic treatment by increasing GLP-1 secretion (13–16), thereby potentially counterbalancing the effects of chemotherapy-induced loss of epithelial cells, possibly including the GLP-1 secreting L-cells (39). The data of the present study, however, does not indicate that such putative mechanism could be adequately effective to maintain sufficient plasma GLP-1 levels in the subgroup undergoing the most intensive myeloablative conditioning therapy, as GLP-1 levels tended to decline after the transplant along with a more pronounced loss of enterocyte mass (shown as more pronounced citrulline decline) and increased inflammation.

Previous studies have suggested the use of GLP-1 as a possible treatment of chemotherapy-induced mucositis (13, 18). Administration of GLP-1 analogs in mice treated with chemotherapy ameliorated mucositis and accelerated healing of the intestinal injury (13, 14). In patients with diabetes, obesity and psoriasis, treatment with GLP-1 analogs has been found to cause reduction in chronic low-grade inflammation (40–43). Although the mechanism is debated and probably confounded by the metabolic-improving effects of GLP-1 treatment (43), amelioration of the putative dysfunctional intestinal barrier might play a role in these conditions (44).

Although the results of the present study point to the relevance of further exploring the clinical potential of GLP-1 to ameliorate side effects of cytotoxic treatment and transplantation, it should be emphasized that GLP-1 treatment is known to cause anorexia and nausea (45), which are already unwanted side-effects for patients receiving chemotherapy. Further investigations including oral glucose tolerance test and clinical trials are needed to evaluate the tolerance of GLP-1 analogs in HSCT patients. Additionally, supplementary data on mucositis scoring and nutritional intake were not available in the present study but would be of interest in future studies.

Previous studies in animals and humans have indicated that GLP-1 is secreted in response to intestinal injury (13, 14, 16). An enhanced GLP-1-secretion may, according to both *in vitro* and rodent studies, be mediated by toll-like receptors at the basolateral sides of the L-cells, which become exposed to endotoxins due to mucosal barrier disruption (16, 46, 47). Such mechanisms might be active in critically ill patients where fasting GLP-1 levels have been found elevated (25, 47). In this study, GLP-1 levels in the patients, including pre-transplant levels, were generally higher than in healthy controls, suggesting that similar mechanisms may, to some extent, be activated already at the timepoint of referral in these often severely affected patients. Our findings, however, appear to be in contrast to a study by Skoczén et al., reporting decreased GLP-1 levels before conditioning in pediatric HSCT patients (48). The reason for this discrepancy is unclear and difficult to judge based on the limited number of participants in their study (27 patients; of those nine had a non-malignant diagnosis) and with an absence of observations in the early post-transplant period.

A strength of the present study is the meticulous design with numerous, consecutive and precise time points for GLP-1 measurements, both pre- and post-HSCT, as well as the inclusion of a large control cohort. Yet, this study is limited by its lack of data on mucositis scoring and nutritional intake, which potentially could have strengthened our conclusions. Moreover, a larger cohort is needed to obtain statistical power to detect possible associations between GLP-1 and risk of inflammatory treatment-related complications, including SOS and aGvHD.

In conclusion, we have presented evidence of an association between endogenous GLP-1 and enterocyte recovery rate and the degree of systemic inflammation in pediatric patients undergoing HSCT. These findings lend support to the notion that GLP-1 may contribute to the well-known large inter-individual variability in the tolerance to chemotherapy and irradiation and suggest new potential therapeutic strategies to prevent toxicities related to chemotherapy in HSCT.

## Data Availability Statement

The raw data supporting the conclusions of this article will be made available by the authors, without undue reservation.

## Ethics Statement

The studies involving human participants were reviewed and approved by the Capital Region of Denmark’s Ethical Committee (H-7-2014-016). Written informed consent to participate in this study was provided by the participants’ legal guardian/next of kin.

## Author Contributions

ME contributed to data collection, performed laboratory and statistical analyses and interpretation and drafted the manuscript. HL, BH, and JH contributed to project design, laboratory analyses and data interpretation. KK and KS established sample collection design and contributed to sample collection. SS, CF-B, CF, J-CH, and TH contributed to sample collection and data interpretation. KM designed the project, established the collaboration and contributed to data interpretation. All authors critically revised the manuscript and gave their final approval of the version to be published.

## Funding

Financial support was obtained from The Research Council at Rigshospitalet, The Childhood Cancer Foundation (#2017-1997), the NNF Center for Basic Metabolic Research, University of Copenhagen and the BRIDGE – Translational Excellence Programme (bridge.ku.dk) at the Faculty of Health and Medical Sciences, University of Copenhagen, funded by the Novo Nordisk Foundation (#NNF18SA0034956).

## Conflict of Interest

The authors declare that the research was conducted in the absence of any commercial or financial relationships that could be construed as a potential conflict of interest.

## Publisher’s Note

All claims expressed in this article are solely those of the authors and do not necessarily represent those of their affiliated organizations, or those of the publisher, the editors and the reviewers. Any product that may be evaluated in this article, or claim that may be made by its manufacturer, is not guaranteed or endorsed by the publisher.
